# Effects of Particle Size of Dielectric and Operating Time through Nonthermal Plasma on Fuel Characteristics of Refined Fuel from Oily Sludge

**DOI:** 10.1021/acsomega.5c03027

**Published:** 2025-10-15

**Authors:** Cherng-Yuan Lin, Ying-Hao Liao, Tzu-Hsuan Hsu, Chiang Fu

**Affiliations:** † Department of Marine Engineering, 34880 National Taiwan Ocean University, Keelung 20224, Taiwan; ‡ Department of Mechanical Engineering, 34914 National Yang Ming Chiao Tung University, Hsinchu City 300093, Taiwan

## Abstract

The refining processes of oil refineries, waste lubricants replaced by maintenance plants of gasoline and diesel vehicles, and mechanical lubricants of various types of ships produce oily sludge with different complex components, including varying amounts of water, hydrocarbons, metal rust, solid residues, inorganic compounds, and other components. The high heavy content of the sludge results in its high kinematic viscosity, and it exists in a liquid state close to a solid state at ambient temperature. Although the composition of oily sludge is complex and difficult to separate, its heating value can still reach 9000 cal/g, which is about 80% of the heating value of gasoline or diesel. Therefore, it has a great recycling value. This study therefore intends to develop oily sludge refining technology and establish an economical treatment process by nonthermal equilibrium plasma. The pretreatment technology of solvent extraction combined with filter filtration was used to extract and separate the oily part of the sludge containing hydrocarbons from the oil sludge. Two types of nonthermal plasma, such as DC streamer discharge and dielectric plasma discharge, were considered for the refining processes. Under operating conditions, the oily sludge is cracked and intensified to produce liquid and gaseous fuel products. The gaseous products are condensed and collected into fuel oil by a vacuum rotary concentrator. The composition and fuel properties of the fuel refined under different operating conditions of nonthermal plasma, including dielectric particle size of Al_2_O_3_ and action time, were tested. The research results showed that the pretreatment procedure can effectively improve the properties of originally discarded sludge at the beginning of the oil treatment process. In the second part of the experiment, after comparing different types of plasma reactors, the direct treatment type of dielectric-barrier discharge (DBD) plasma was found to be superior because the plasma can directly contact the treated sludge and extended the plasma treatment range. When quartz glass beads were added to the sludge, a large number of raised areas were formed on the surface of the sludge, making it easier to form a tip discharge for plasma generation and increasing the plasma treatment area. When the weight ratio of dielectric to sludge, the operating time, and the particle size were 2/1, 8 min, and 100 μm, respectively, there is the highest heating value and the lowest residual carbon content. In addition, the smaller the dielectric particle size, the smaller the carbonaceous size of the refined oil after the plasma treatment. As the plasma treatment time increased, the surface of the oily sludge was carbonized, resulting in a reduction in the plasma operating range. This research converts waste sludge into a fuel of precious energy, thus providing an important contribution to the development and application of energy.

## Graphical abstract

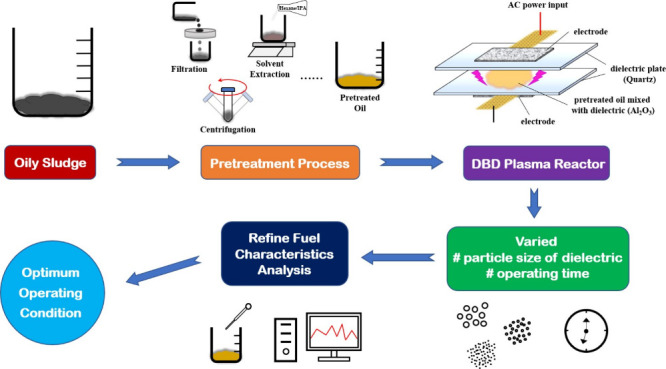

## 1 Introduction

A large amount of oily sludge is produced during the refining, mining, production, storage, and use of petroleum products, including the lubricant industry chain used in transportation and industry. Sludge often accumulates at the bottom of storage oil tanks, usually in solid or semisolid form. Its components include petroleum hydrocarbons and other components that are difficult to handle, such as mud, sand, water, metal scrapings, inorganic compounds, rust, ash, etc. The sources of sludge include lubricating grease from internal combustion engines and external combustion engines used in transportation, waste lubricating oil replaced by automobiles and locomotives, waste engine oil, and grease used in steel mills or refineries.^1,2^ After preliminary analysis, the sludge produced at the end of the waste lubricating oil treatment process contains approximately 20–70 wt % hydrocarbons, 5–70 wt % water, and 20–50 wt % inorganic solid compounds.^3^ The sludge contains a heating value of about 6000–8000 cal/g. Therefore, through appropriate treatment methods and refining technology, the waste from the sludge process can be converted into valuable energy resources to produce fuel oil used in industrial boilers or gasoline or diesel used in locomotives. On the contrary, oil sludge is regarded as industrial waste. If it is dumped into rivers or soil without proper treatment, it will cause irreversible damage to the biological environment and harm to human health.^4–6^

Plasma is the fourth state of matter and can be regarded as an ionized gas. The positive and negative ions and electrons in it can move freely and are conductive, unlike neutral gases that are generally nonconductive. Plasma can be generated by applying an external electric field. When the electric field is strong enough to cause an electron avalanche, plasma can be generated. Plasma contains positive and negative ions, electrons, and neutral gas molecules. These particles of various types and characteristics are the reason plasma can be widely used in different fields. For example, high-energy electrons in plasma can stimulate many chemical reactions by colliding with other particles; ion bombardment is used in semiconductor manufacturing processes, free radicals can be used in material surface treatment, and excimer molecules are used in luminescence or lighting applications. If plasma is classified by temperature, it can be divided into low-temperature plasma (LTP) and high-temperature plasma (HTP).^7^ Among them, according to the energy balance state between particles, low-temperature plasma is divided into thermal equilibrium plasma and nonthermal plasma.

As the name suggests, thermal balance plasma means that the temperatures of electrons, ions, and neutral gas molecules reach thermal equilibrium. Common types of plasma include arc, spark plug discharge, and plasma welding, all of which belong to the thermal equilibrium plasma. In nonthermal equilibrium plasma, the gas dissociation rate is not high, and the electron density is also lower than that of thermal equilibrium plasma. The temperature of the background neutral gas molecules and ions is much lower than the electron temperature. Both streamer discharge and dielectric-barrier discharge (DBD) belong to this type of plasma.^8^ In nonthermal equilibrium plasma, due to the low gas dissociation rate, the background gas temperature can be controlled close to room temperature, so it can directly contact temperature-sensitive experimental objects. This type of plasma is often used in medical, biological, and agricultural applications. As described in the previous introduction to plasma, plasma has a wide range of application fields, including aerospace, energy, materials, lighting, food processing, agriculture, waste treatment, biomedicine, etc. The scientific research on plasma in these fields is also extremely new and popular. In fact, low-temperature plasma has been used in these application fields for a long time and is even regarded as an enabling technology, but limited attention has been paid.

Compared with thermal equilibrium plasma, nonthermal equilibrium plasma has been more commonly used in combustion applications and research in recent years. Corona discharge, streamer discharge, radio frequency (RF), microwave (MW), and nanosecond pulse discharge (NSD) are all nonthermal equilibrium plasmas.^9^ Although the gas dissociation rate and electron density of nonthermal equilibrium plasma are lower than those of thermal equilibrium plasma, its electron temperature or energy is relatively high (1–100 eV, 1 eV is approximately equal to 11,604 K). High-energy electrons collide with other particles can more effectively carry out excitation, decomposition or ionization reactions, producing free radicals or active particles required for combustion reactions.^10^ Since these chemical reactions accompanying electron collisions are affected by the energy of the electrons, the combustion gain of nonthermal equilibrium plasma depends on the physical properties of the plasma, such as the temperature and density of electrons in the plasma.

There are many traditional ways to treat sludge, which can basically be divided into thermal treatment (such as direct incineration), physical treatment (such as filtration and separation), chemical treatment (such as extraction and solidification with chemical additives), or biological reaction. Although these methods have industrial applications and can effectively treat sludge, each method has its shortcomings. For example, although direct incineration can greatly reduce the volume of sludge waste and the heat produced from the combustion process can be used as renewable energy, the ash and waste gas generated by its combustion may still contain dioxin components and additional waste gas treatment processes are required to reduce environmental impact. Another example is burying oil sludge. Although some components of oil sludge can be decomposed through biochemical reactions, direct burial will have a huge impact on soil, water sources, and crops and even cause harm to the human living environment.

Basically, most of the current literature uses thermal plasma reactors to treat oil sludge. The thermal plasma treatment process can be roughly divided into two stages^11^: (1) thermal conversion process, this stage is the phase change process, such as the evaporation of volatile substances; (2) thermal decomposition process, this stage is mainly related to high-temperature autonomous chemical reactions and plasma-induced chemical reactions. These processes may oxidize and crack sludge or vaporize organic substances and vitrify inorganic compounds. The technologies related to plasma treatment of sludge in the current research literature include DC plasma arc, radio frequency plasma (RF plasma), microwave plasma (microwave plasma), etc., almost all of which are thermal balance plasma technologies. These technologies mainly use combustion, pyrolysis, and gasification to achieve the purpose of sludge treatment. Different plasma technologies have different processing advantages. For example, radio frequency plasma can evaporate heavy metal components in sludge more efficiently than DC plasma, and DC transferred plasma arc can evaporate heavy metal components in sludge more efficiently than DC nontransferred plasma arc. Nontransferred plasma arcs can more effectively vitrify sludge. In addition, the design of the plasma reactor will also affect the conversion and decomposition efficiency of the sludge. Striu̅gas et al.^12^ pointed out that the length of time for plasma treatment of sludge will affect the tar content in the sludge. The power of the plasma will also affect the conversion composition and quality of sludge. Taking DC plasma arc as an example, Ali et al.^13^ pointed out that when the power of plasma arc is increased, the total organic carbon (TOC) in the sludge can be effectively reduced. TOC content increases the higher heating value (HHV) of the flue gas, improves the energy yield of the flue gas, and changes the chemical composition of the flue gas. Ali et al.^13^ also found that after treating sludge with thermal plasma, if the flue gas contains methane, ethane and acetylene, it will have a higher heating value. However, if the plasma power is too high, part of the sludge will be converted into carbon monoxide and hydrogen and the heating value of the flue gas decreases, accompanied by an increase in a large amount of nitrogen oxides (NOx). The power of the plasma arc will also affect the vitrification results of the sludge. The higher the plasma power, the higher the propensity it can convert the sludge to solid glass.

Based on the review and analysis of the above important relevant literature, we understand that the sludge produced by oil refineries, steel mills, automobile and motorcycle lubricants, and lubricating greases used in industrial processes has a high heating value, high viscosity, and the ability to remove PHCs (petroleum hydrocarbons). In addition to the oil components, there are varying amounts of impurities, asphaltenes, water, metal compounds, and other components. It is a useful resource that can be refined into boiler fuel oil or gasoline and diesel for vehicles. By refining recycled sludge, it is one of the effective ways to practice circular economy. A plasma reactor will be set up to refine the sludge of waste lubricating oil into fuel and conduct various fuel properties tests of the sludge before, during, and after refining. It will also use a diesel engine to test and analyze the engine power and exhaust characteristics for the refined sludge.

In addition to the design of the plasma reactor itself and the plasma power, other physical parameters that are very important to the sludge conversion process include plasma temperature, working gas, and sludge raw material composition. The influence of the plasma temperature is mainly due to differences in chemical reaction pathways and reaction rates. Although higher-temperature plasma is beneficial to the reduction of the volume and mass of sludge and is also conducive to the conversion of total organic carbon, this does not mean that higher temperature plasma favors overall sludge conversion. The product composition of the flue gas affects the lower heating value (LHV) of the sludge after conversion. If the working gas of the plasma is air, excessive temperature will affect the overall energy recovery.^14^ At the same time, the plasma temperature will also affect the volatilization degree of the solid particles and metal debris contained in the sludge. Since plasma is an ionized gas, different working gases not only affect the plasma starting voltage and working power but also produce excited particles and chemical free radicals of different energies, which in turn affect different conversion reactions and produce different products and flue gas heat. Air is one of the most common working gases for plasma, mainly because air is easy to obtain and has a low economic cost. However, oxygen in air easily reacts with flammable volatile gases in sludge. Although it can produce syngas fuel, many polluting gases are produced meanwhile, such as nitrogen oxides (NO and NO_2_) and sulfide oxides such as SO_2_.^1,15^ In recent years, inert gases such as argon or nitrogen were used to conduct research for cracking sludge by plasma,^16^ but the relevant research is still quite limited, and most plasma technologies used are based on thermal equilibrium plasma.^17^

Peeters and Butterworth^18^ used an oscilloscope to view the power (*Q*) and voltage (*V*) of the Lissajous graph. The *QV* graph was used to calculate the electrical energy required for DBD plasma and then derived the energy efficiency of DBD plasma. The ideal *QV* pattern will be a parallelogram, and the *QV* pattern presented by the actual DBD plasma will be affected by the amplitude of the operating voltage. The air in the dielectric during discharge and the frequency of the operating voltage form an approximately almond-shaped parallelogram. First, the current *i*_m_(*t*) flowing through the monitor needs to be converted into *i*(*t*). For example, eq 1 uses the capacitance and the voltage that change with time to calculate *i*(*t*). Next, the instantaneous DBD plasma power is the product of voltage and current. Finally, the voltage that drives plasma is alternating current, which can prove that the intensity of plasma is related to voltage and frequency. Miao et al.^19^ found that nonthermal plasma can generate free radicals which are recombined to remove impurities or pollutants. For example, the removal of NO and SO_2_ in exhaust gas has achieved great success, and other nitrogen oxides and sulfur oxides can also be destroyed, converted, and reorganized through the catalytic process of nonthermal plasma. Combining DBD with different catalysts can further improve its ability to remove impurities or pollutants. Nonthermal plasma can also destroy, transform and recombine volatile organic compounds, such as the oxidation of benzene (C_6_H_6_) to produce phenols.^20^

The high-energy electrons and active particles released by plasma discharge can directly or indirectly change the chemical reaction pathway of the reacting substances, thereby promoting the cracking of substances and the process of distilling volatiles with lower boiling points. The process has a high chemical reaction efficiency and heat transfer. Rapid, green, and clean process and other benefits occurred. However, there are no literature that used nonthermal plasma to refine sludge into liquid fuel.^21^ Although nonthermal plasma has been commonly used in practical applications and academic research in the general combustion field. Nanosecond pulsed discharge (NSD), radio frequency discharge (RF), corona discharge, etc. all belong to the category of nonthermal plasma.^9^ Compared with thermal equilibrium plasma, the ion temperature, electron density, and gas dissociation rate of nonthermal equilibrium plasma are lower, but its electron energy and temperature are relatively higher. Through the collision of high-energy electrons with particles of the reacting material, it can effectively promote ionization, decomposition, or excitation reaction to produce active particles or free radicals that crack sludge and other substances. Although thermal plasma technology has been used in solid waste treatment, there is still a lack of research on the plasma conversion of sludge. The process and benefits of low-temperature pyrolysis of sludge are still unknown. In addition, compared with thermal equilibrium plasma, nonthermal equilibrium plasma consumes less energy and contains high electron energy. The overall plasma temperature is much lower than that of thermal equilibrium plasma, but it is enough to carry out organic cracking reactions and avoid some unnecessary high-temperature chemicals reaction. Hence, it has higher economic value and conversion efficiency for sludge treatment by nonthermal equilibrium plasma. Therefore, this study proposes to use nonthermal equilibrium plasma to treat oil sludge by cracking in an aerobic environment. The plasma reactor used in this study is dielectric-barrier discharge (DBD) and uses AC power to discharge a nonthermal equilibrium plasma. Dielectric plasma uses stainless steel as the electrode. The dielectric material can be quartz or ceramic. This plasma reactor produces a filament discharge. Due to the nonconductive properties of the dielectric, it can prevent the generation of arcs. The method of treating sludge was operated in a chamber with controllable working gas to carry out the sludge cracking reaction. When the working gas is changed to air, the aerobic oxidation reaction is converted. The dielectric plasma reactor in Figure 1 can produce a large area of plasma discharge and is suitable for processing large amounts of sludge. The actual benefits of nonthermal equilibrium plasma for sludge conversion can be explored.

## 2 Experimental Details

The composition of sludge contains varying amounts of oil, water, compounds such as metal shavings, sand, and other solid substances. Preprocessing for the sludge to remove those impurities is required first. The retrieved oil sludge underwent a pretreatment process of solvent extraction and centrifugal treatment in an oil purifier and then used dielectric-barrier plasma discharge (DBD) to carry out cracking and excitation reactions to produce gaseous and liquid products. The gaseous products were condensed and collected into liquid oil through low-temperature cooling in a vacuum rotary concentrator. The collected product was then analyzed for its compositions and fuel properties. The experimental details ​are described below.

### 2.1 Pretreating Sludge Using Oil Extraction by Solvent

Waste lubricants were left after replacement with fresh lubricating oil by automobile and motorcycle maintenance plants, various types of ships, steel mills, and other relevant sources. The sludge remaining at the bottom of the oil tank is often in a semisolid or solid state. Some of the sludge can still flow, but it is generally highly viscous and contains a large amount of water, inorganic compounds, metal rust chips, sand, other solid substances, and residues. Therefore, before using it for further plasma treatment process, it needs to undergo a series of pretreatment steps, such as sedimentation, centrifugation, and filtration, and solvent extraction in order to improve oil properties, increase heating value, and reduce residual carbon content. In this study, a nontoxic hexane was used as the solvent because it has a small impact on the environment to extract the grease in the sludge. The sludge and hexane were put into a beaker, which was stirred and heated by an electromagnetic heating stirrer (Alexdigital model, Velp Scientific, Usmate, Italy). After heating the sludge mixture to 60 °C and maintaining stirring for 20 min, a vacuum rotary concentrator was used to evaporate and condense the hexane solvent for reuse, leaving behind the refined oil (mainly hydrocarbons) in the flask. Rather little oil content with close boiling points to the hexane solvent was entrained with the hexane solvent to leave the refined oil, resulting in the possible low oil loss from the oily sludge.

### 2.2 Set-Up a Nonthermal Plasma for Refining Sludge

A dielectric barrier discharge (DBD) type of nonthermal plasma was set up to carry out the cracking and refining reaction of the sludge to produce fuel. The dielectric barrier discharge (DBD) nonthermal plasma used AC power to produce mercerized discharge.^22^ This study therefore used one high-voltage DC power supply and one high-voltage AC power supply. The schematic diagram of the experimental configuration of converting sludge by a nonthermal plasma is shown in Figure 1. The nonthermal plasma reactor acted on sludge in an aerobic operating environment. In addition, the dielectric particle sizes and operating times were controlled to test their effects on oil properties of products obtained from refining sludge.

**Figure 1:**
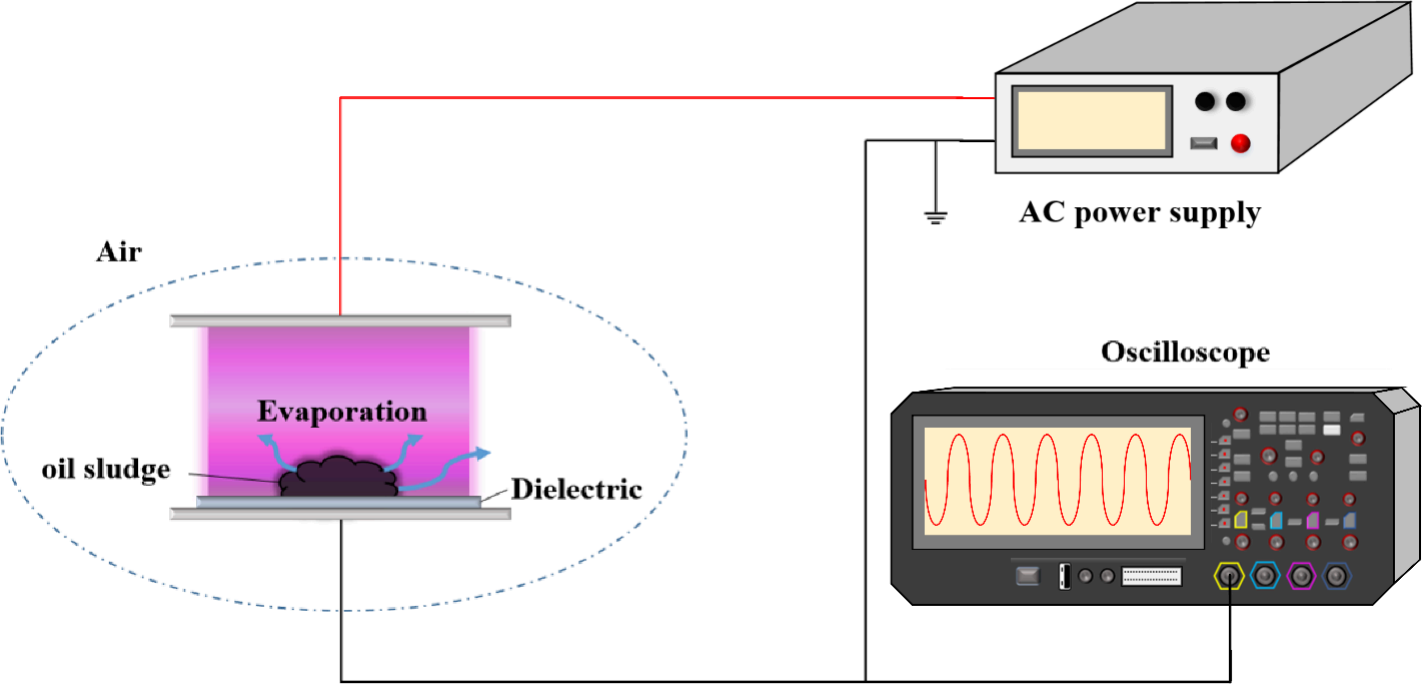
Experimental configuration of using a dielectric barrier plasma device to treat sludge.

The electrodes are made and configured by attaching a 5 cm square stainless-steel mesh as a high-voltage electrode on top of the quartz plate. In addition, the same piece of stainless-steel mesh is fixed on the flat plate as a ground electrode. Both high-voltage and ground electrodes use a 325-mesh stainless steel mesh. In order to facilitate the connection of the power supply output line, additional copper foil tape is used to stick to the surface of the mesh to extend the conductive area. The distance between the two electrodes is 3 mm, and dielectric material which was a quartz sheet with a thickness of 1 mm was used. 1 g of sludge evenly mixed with quartz glass beads with a diameter of 1 mm was prepared as the sludge sample. The distance between the sludge surface and the quartz sheet is less than 1 mm, which is very close and thus theoretically can reduce the voltage required to form plasma. The uniformity of the slurry ultimately increases the overall processing efficiency. When the power supply is turned on, plasma will be generated under the quartz plate and in the gap between the sludge surface and the quartz plate. The appearance changes before and after treatment was recorded by the camera.

The detailed steps of dielectric plasma discharge in this experiment are described as follows: (1) sample preparation: the dielectric Al_2_O_3_ of different particle sizes (10, 100, 1000 μm) was added to the pretreated sludge. The sludge mixtures were sufficiently stirred and mixed by an electromagnetic stirrer. The weight ratio of the sludge to the dielectrics was set at 2:1 and 4:1; (2) setting the plasma discharge frequency and voltage: using an AC power supply to adjust the discharge frequency and using an oscilloscope to check the voltage and confirm the frequency. The frequency range supplied is 15–25 kHz, and the maximum supply voltage is 10 kV; (3) plasma acting on the sludge: turn on the high-voltage AC power supply, programmable switching DC power supply, and oscilloscope, respectively, and connect the wires and alligator clips between the devices to ensure that all devices can operate normally. Take an appropriate amount of the prepared sample and spread it evenly on the action area of the DBD plasma reactor, trying to ensure that the sample does not touch the upper glass or overflow the plasma action area. Then the current output switch was turned on, the current slowly increased to 80 mA, and we observed whether the area generated by the plasma covers most of the action area. The blue-violet light area is the range of plasma action. The action time of the plasma was set from 30 s to 8 min. After completion, take out the sample and place it in a centrifuge tube; (4) aftertreatment of the sludge sample: since the sample after plasma treatment contains dielectric particles and carbon particles produced during plasma treatment, the sample needs to be centrifuged again. The centrifuge was turned on to rotate at high speed (2000–6000 rpm) for at least 3 min to separate the components of different specific gravities in the sample. The impurities with higher density are deposited in the lower layer of the test tube, while the oil with lower density floats in the upper layer of the test tube and then takes out by using a clean dropper to suck out the upper oil and store it in a clean test tube.

### 2.3 Analysis of the Fuel Sample Properties after Nonthermal Plasma Refinement

The fuel oil which was extracted from the sludge through thermal cracking by the DBD nonthermal plasma was analyzed its compositions and fuel properties.

The elemental compositions of the fuel sample such as C, H, and O were analyzed with a CHN-OS Rapid Elemental analyzer (model Vario EL, Heraeus Ltd., Langenselbold, German). The chemical compounds of the derived fuel sample were analyzed by gas chromatography associated with a mass spectrometer (GC/FID, model Agilent 8860, Agilent Technologies Ltd., Santa Clara, CA, USA). The length, width, and thickness of the internal column (model HP-5, Agilent Technologies Ltd., Santa Clara, CA, USA) used for the analysis were 30 m, 0.32 mm, and 0.25 μm, respectively. A volumetric cartridge moisture titrator (model 870 KF Trino plus, Metrohm AG, Switzerland) was used to measure the moisture content in the sample based on the amount of titrant consumed by dripping the sample. The moisture unit can be expressed in weight percentage or ppm. The heating value of the fuel sample was analyzed by an oxygen-bomb automated adiabatic heat calorimeter (model 6772, Parr Instruments Ltd., Moline, IL, USA) with a temperature resolution of 0.0001 °C. It contains a microprocessor and a constant temperature water tank, which can accurately measure the temperature rise after the combustion of fuel sample. The higher heating value of fuel means that its unit mass can release higher heat after combustion reaction and can thus have larger engine horsepower output. The carbon residue indicates the weight percentage of the carbon content left after the burning of the oil sample to the original weight of the fuel prior to its burning in the adiabatic calorimeter. A Fourier transform infrared spectrometer (Model Tensor II, Bruker Company, Ettingen, Germany) was used to analyze the chemical composition of the refined oil products. The Raman scattered infrared spectra was acquired by the FTIR to determine the oil compositions.

The indirect treatment design of the DBD plasma reactor has a circular copper piece with a diameter of 3 cm bonded above the quartz plate as a high-voltage electrode. The stainless-steel screen is immediately below the quartz plate, and the screen is used as a ground electrode. The sludge of 10 g was poured into a Petri dish and placed under the screen for processing. When the power supply was turned on, plasma was generated under the quartz plate and in the gap between the metal screens. The high-energy free radicals generated by freeing air through plasma action will be processed indirectly on the sludge. In order to facilitate the connection of the power supply output line, additional copper foil tape was used to stick to the surface of the mesh to extend the conductive area. The distance between the two electrodes is 3 mm, and the dielectric material of a quartz sheet with a thickness of 1 mm was used. The sludge was evenly mixed with quartz glass beads. The dielectric material of quartz glass beads of various sizes was added to the treated sludge. When the power supply was turned on, plasma was generated under the quartz plate and in the gap between the sludge surface to treat the sludge.

## 3 Results and Discussion

The sludge does not contain dielectrics, so adding dielectrics of quartz glass beads can effectively reduce the collapse voltage of the triggered plasma. Two parameters in the plasma, namely, dielectric particle size varying from 1000 to 10 μm and operating time from 8 to 0.5 min, were adjusted. The results of the fuel properties of the treated sludge were obtained and discussed. At least 3 times were repeated for each measurement to obtain the mean values. The uncertainties of the carbon residue, water content, and heating value were ± 2.16%, ± 1.23%, and ± 1.57%, respectively.

### 3.1 Heating Value

Heating value is an important indicator of fuel quality. The higher the heating value of fuel, the better its combustion performance is. The calorific value affects the efficiency of fuel use and fuel economy. Fuel with a higher heating value has more heat energy and can more effectively drive transportation equipment. From an economic perspective, using fuel with a high heating value can effectively reduce fuel usage. The weight ratio of dielectrics Al_2_O_3_ to oily sludge was set at 1/2. The effects of the plasma operating time on the heating value of the refined fuel oil are shown in Figure 2. The surface of the oil was found to form a black solid layer which is visible to the naked eye. In addition, it was also observed during the plasma operation, the range of plasma treatment of sludge decreased as the operating time increased. After 8 min of plasma operation, only the corner of the plasma operation range was left. Hence, the longest operating time was set 8 min.

**Figure 2:**
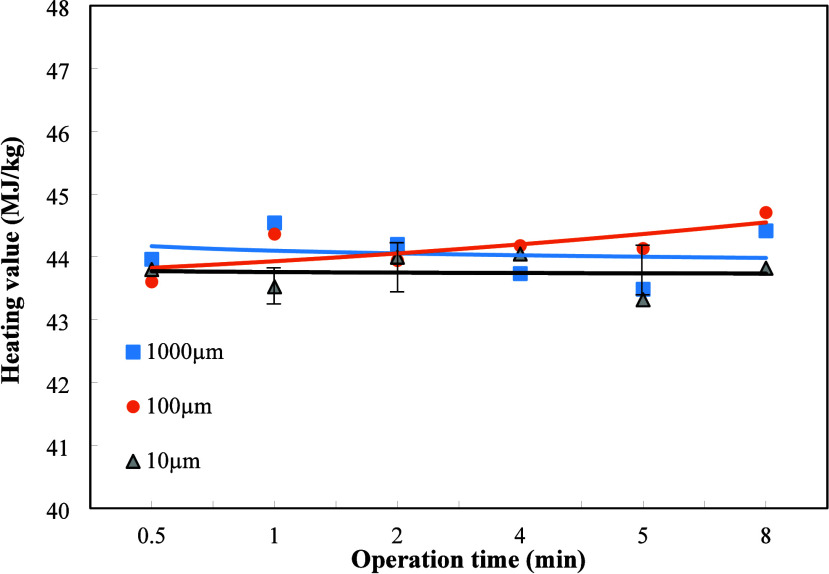
Effects of dielectric particle sizes and plasma operating treatment times on the heating value of refined oil when the weight ratio of Al_2_O_3_ and oily sludge is 1/2.

The increase of operating time was observed to increase the heating value of the refined oil for the dielectric particle size of 100 μm, which trend is adverse to that of the dielectric particle size of 10 μm. For the dielectric particle size of 1000 μm, the increase of operating time was shown to increase and decrease the heating value. The higher heating value occurred at the operating times of 1 and 8 min, which reached 44.5 MJ/kg for the dielectric particle size of 1000 μm. However, for the dielectric particle size of 10 μm, the refined oil had relatively lower heating value, 43.5 MJ/kg at the same plasma operating time of 1 min.

When the plasma operating time was less than 4 min, a larger dielectric particle size caused a higher heating value. Hence, the dielectric particle size of 1000 μm resulted in a higher heating value of 44.5 MJ/kg after 1 min of plasma operating. The larger dielectric particle size such as 1000 μm reduces the distance between the sludge surface and dielectrics quartz, leading to trigger plasma at lower voltage. Therefore, the dielectric particle size of 1000 μm caused the larger extent of plasma action, leading to higher heating value of the refined oil under shorter operating time less than 4 min. After 4 min, the dielectric particle size of 100 μm turned out to have the highest heating value among those three particle sizes under 8 min of plasma operating time. This is due to the fuel oil temperature increasing with the increase of the plasma operating time. The kinematic viscosity of the oil was reduced accordingly to cause part of the refined oil to fly out from the plasma operating area. The remaining fuel oil was carbonized to leave more carbon residue. Finally, the larger dielectric particle size caused the decrease of heating value, and the dielectric particle size of 100 μm became the adequate particle size to have the most powerful plasma operation. The smallest dielectric particle size of 10 μm still had too far distance between the sludge surface and dielectrics quartz, leading to the lowest heating value among those three particle sizes, as shown in Figure 2.

Figure 3 shows the variation of heating values of the oil after treatment with different dielectric particle sizes and plasma treatment times when the weight ratio of dielectric Al_2_O_3_ to the sludge is 4/1. Under various dielectric particle sizes, the heating value of the oil decreases with the increase of plasma operating time. It is ascribed to that the plasma action is affected by the spacing size in the plasma reactor^23^ and the size of the dielectric particle.^24^ The weight ratio of dielectric to sludge was set at 4/1, and the particle sizes were varied from 10 to 1000 μm. The results in Figure 3 appeared to have the highest heating value for the minimum particle size of 10 μm. On the contrary, when the dielectric particle size is up to 1000 μm, the heating value was shown to be in an opposite trend and the lowest ones.

**Figure 3:**
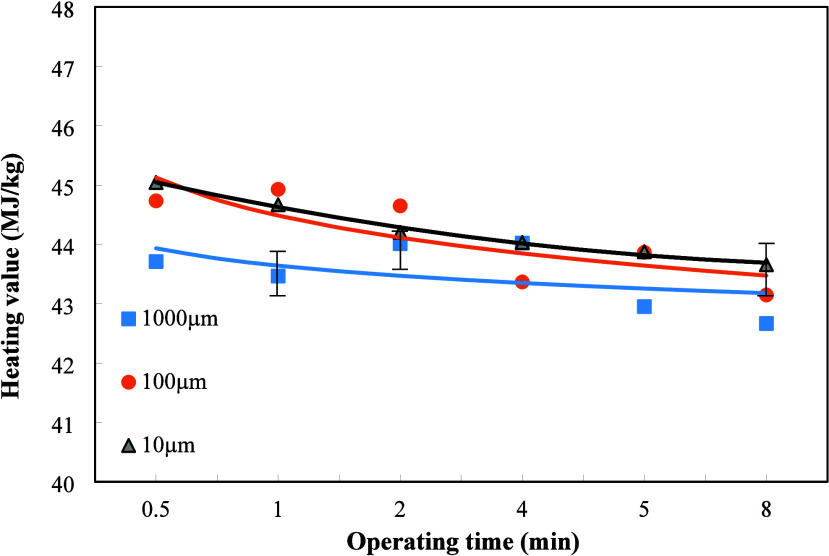
Effects of dielectric particle sizes and plasma operating time on the heating values of the refined oil when the weight ratio of Al_2_O_3_ to oily sludge was 4/1.

Therefore, it can be found that the effects of the weight proportion of dielectric to sludge in the reactant had an inverse relationship with the particle size and also seriously affected the oil quality. In addition, the increase in the operating time resulted in the decrease in the heating value for those three different particle sizes. In comparison with those results of the weight ratio of dielectric to sludge of 2/1 in Figure 2, the current results for the weight ratio of 4/1 are adverse. The smaller dielectric particle size caused higher heating value, implying more successful plasma reaction for the sludge^24^ under larger weight ratio of dielectric to sludge and thus shorter distance between the sludge surface and dielectrics.

### 3.2 Carbon Residue

Carbon residue indicates the extents of fuel quality and combustion efficiency. The amount of carbon residue is generally inversely proportional to the heating value of the fuel oil. Moreover, higher carbon residues occurred with deteriorated combustion efficiency and more serious pollutant emissions, leading to increased environmental impact.

The weight ratio of dielectric Al_2_O_3_ to the sludge was set at 2/1. The amount of carbon residue appeared to increase with the increase in the plasma operating time for the dielectric particle size of 1000 μm in Figure 4. This is due to that uneven plasma action resulted in the increase of the amount of carbonization of the oil with the increase of the plasma operation time and thus increased the carbon residue. In contrast, for the particle size of 100 μm, the amount of carbon residue decreased with the increase of the plasma operating time. This is ascribed to the fact that the plasma reaction moved toward more complete under the adequate particle size of 100 μm, leading to a greater extent of oil refinement and less formation of carbon residue in Figure 4. In comparison with Figure 2, the heating value increased, while the carbon residue decreased with the increase of the operating time for the particle size of 100 μm. The carbon residue first increased and then decreased with the increase of the operating time for the particle size of 10 μm. This is because that incomplete plasma reaction at the beginning of plasma action, the increase of operating time made the plasma action reached a higher complete extent and thus decreased carbon residue.

**Figure 4:**
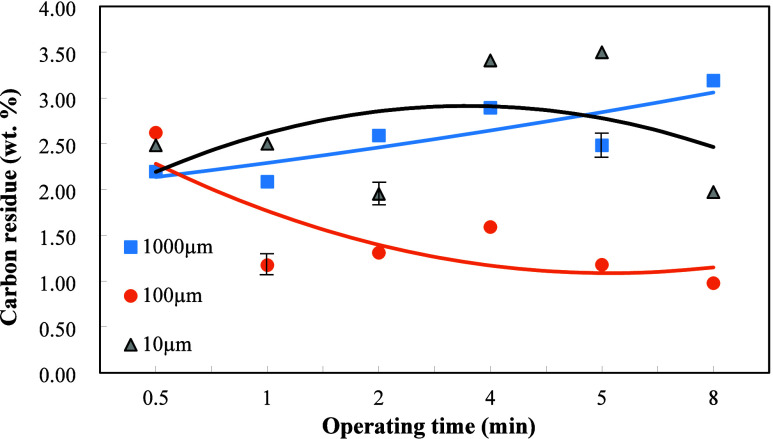
Effects of dielectric particle sizes and plasma operating time on the carbon residue of the refined oil when the weight ratio of Al_2_O_3_ to oily sludge was 2/1.

Figure 5 shows the carbon residue produced under different dielectric particle sizes and plasma operating times when the weight ratio of dielectric Al_2_O_3_ to the sludge was 4/1. The carbon residue was shown to decrease with the increase in the plasma operating time for the dielectric particle size of 1000 μm. This is due to the extent of oil refinement increasing with the increase of the plasma operating time with the largest dielectric particle size of 1000 μm. The decrease of the dielectric particle size down to 100 μm caused an increase of carbon residue with the plasma operating time. This is owing to the increase of plasma operating time caused more carbonization of the treated sludge and thus a larger amount of carbon residue. The even smaller dielectric particle size to 10 μm resulted in first decreased and then increased carbon residue with the plasma operating time in Figure 5.

**Figure 5:**
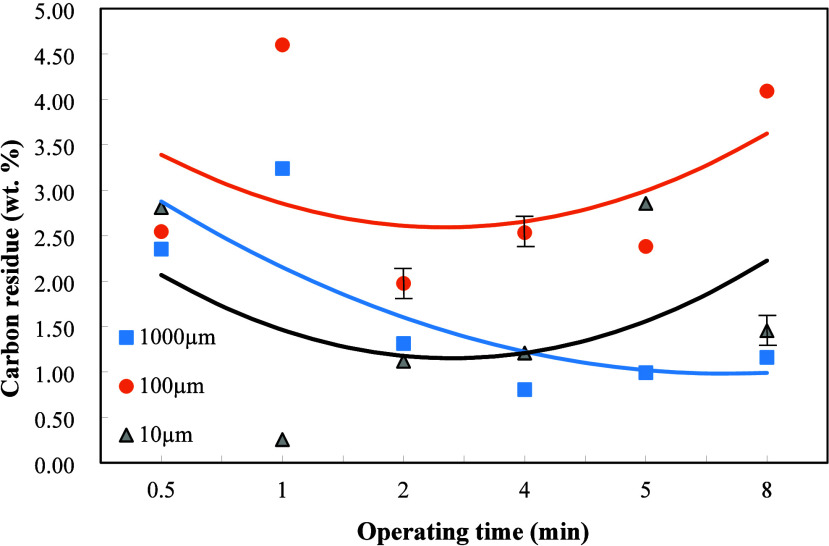
Effects of dielectric particle sizes and plasma operating time on the carbon residue of the refined oil when the weight ratio of Al_2_O_3_ to oily sludge was 4/1.

The results are presented for particle sizes of 100 and 10 μm. The residual carbon amount decreases with the increase in plasma treatment time. Corresponding to the calorific value diagram of the same proportion, it can be found that the two curves show an inverse relationship, which also once again verifies the relationship between the calorific value and residual carbon amount. The lower dielectric particle size of 10 μm facilitated the extent of fuel refinement through the plasma reaction, leading to a smaller amount of carbon residue. The even increase of the plasma operating time rendered further carbonization on the sludge surface,^23^ and thus more formation of carbon residue.

### 3.3 Moisture Content

The moisture content in the oil affects the heating value of the fuel,^25^ combustion efficiency, metallic pipeline corrosion, and NOx formation. Oil with high moisture content directly reduces its heating value and burning temperature and lowers the degree of complete burning. The liquid vapor produced from the burning of water might corrode the exhaust pipe and the internal combustion engine cylinder. Figure 6 shows the effects of dielectric particle size and plasma operating time on the moisture content when the weight ratio of dielectric Al_2_O_3_ to sludge was 2/1. When operating plasma with particle sizes of 1000 and 10 μm, the trends of variation of moisture content with the plasma operating time are roughly similar. As the plasma acts on the mixture of oil and dielectric, it is expected that the moisture content decreased with the increase of plasma operation time, for the cases of particle sizes of 1000 and 10 μm. There is an opposite trend when the particle size is 100 μm. This might be because atmospheric plasma was carried out to inhale more environmental moisture than the water evaporation away by the plasma reaction, leading to increasing moisture content with the plasma operating time for the dielectric particle size of 100 μm as shown in Figure 6.

**Figure 6:**
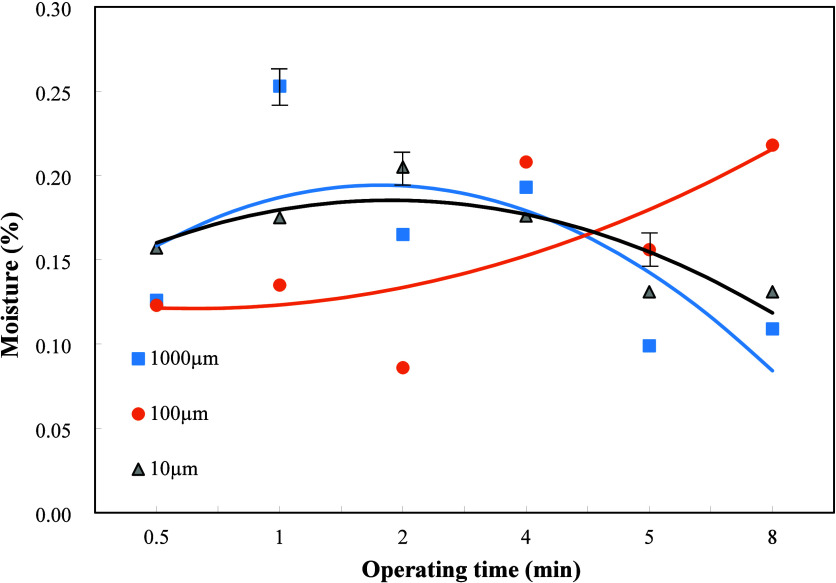
Effects of dielectric particle sizes and plasma operating time on the moisture content of the refined oil when the weight ratio of Al_2_O_3_ to oily sludge was 2/1.

Figure 7 shows the effects of dielectric particle sizes and plasma operating times on the moisture content when the weight ratio of dielectric Al_2_O_3_ to sludge is 4/1. The moisture contents for those three different particle sizes have similar curve trends. The plasma action facilitated the chemical reaction as shown in eqs 1 and 2, in which water is dissociated to form OH and H radicals. The dielectric particle size of 100 μm was shown to have the highest water content, which decreased slightly with the increase in plasma operating time. In contrast, the dielectric particle sizes of 10 and 1000 μm caused the increase of moisture content with the plasma operating time. However, the moisture content at the longest plasma operating time of 8 min of water was still lower than that of the dielectric particle size of 100 μm. In comparison with the carbon residue result in Figure 5, the highest carbon residue appeared for the dielectric particle size of 100 μm. This implied that the most incomplete plasma reaction occurred for the dielectric particle size of 100 μm, leading to the highest carbon residue and the least water dissociation by the plasma action^26^ and thus the highest water content among those three dielectric particle sizes under the weight ratio of Al_2_O_3_ to sludge equal to 4/1. In contrast, for the dielectric particle size of 10 μm, the lowest carbon residue and moisture content were formed, which implied the highest extent of plasma reaction for the dielectric particle size.

$$H_{2}O⇌H+\mathrm{OH}$$ 1

$$H+H_{2}O⇌H_{2}+\mathrm{OH}$$ 2

**Figure 7:**
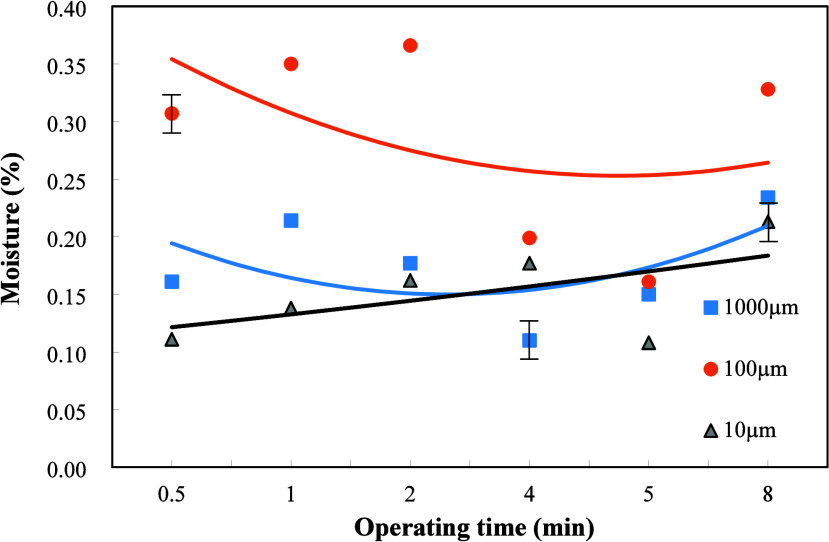
Effects of dielectric particle sizes and plasma operating time on the moisture content of the refined oil when the weight ratio of Al_2_O_3_ to oily sludge was 4/1.

### 3.4 Analysis of Fourier Transform Infrared Spectroscopy (FTIR)

The analysis of Fourier transform infrared spectroscopy has many advantages, including multiple wavelength scanning, high sensitivity, high accuracy, and high resolution. The principle of the instrument is to analyze the infrared absorption by utilizing the characteristics of functional groups in compounds that absorb infrared rays of specific wavelengths. Spectra are determined by identifying the functional groups contained in the sample through Fourier transform operation so as to conduct qualitative or quantitative analysis.

Figure 8 shows the Fourier transform infrared spectra for the pretreated oil and the oil acted by the plasma with dielectric particle size 1000 μm and the weight ratio of dielectric to sludge equal to 4/1, under various plasma operating times ranging from 30 s to 8 min. From the transmission spectrum obtained by Fourier transform infrared spectroscopy analysis of two different oil samples, it can be seen that the lowest transmittance is at a wavelength of 2921 cm^–1^. From the spectra, it is judged that there is symmetrical CH_3_ stretching in the sample. At the wavelength of 2853 cm^–1^, the symmetrical CH_2_ stretching existed. At other wavelengths such as at the wavelength 1741 cm^–1^, it is aldehydes containing carbon–oxygen double bonds; at the wavelength 1460 cm^–1^, it is the CH_3_ vibration, and at the wavelength 1377 cm^–1^, it contains CH_2_ vibration. The other two wavelengths are 1065 and 875 cm^–1^. The former is alcohol containing carbon–oxygen bonds.^27^ Alcohols are oxygen-containing substances. Their presence in oil can cause more full burning during engine operation. The latter are aromatic hydrocarbons containing carbon–hydrogen bonds. Their presence in oil can increase the density and heating value of the oil. However, aromatic hydrocarbons can easily produce carbon deposits and polycyclic aromatic hydrocarbons (PAHs) in engines after burning. When absorbed by the human body, PAHs will change their DNA structure, leading to an increase in the rate of genetic malformations and an increase in the risk of cancer.^28^ If released into the environment, they will contaminate soil, water sources, animals, and plants. etc., which have adverse effects on the environment. Hence, from a practical perspective, their presence in oil products should be avoided as much as possible. Based on above explanation and the experimental results shown in Figure 8, it can be known that the regenerated oil obtained from the plasma treatment for 8 min has a higher alcohol content and a lower aromatic content. If based on the transmission spectra obtained from Fourier transform infrared spectroscopy analysis, it can be inferred that the longer the plasma operating time, the superior oil quality that plasma action can improve.

**Figure 8:**
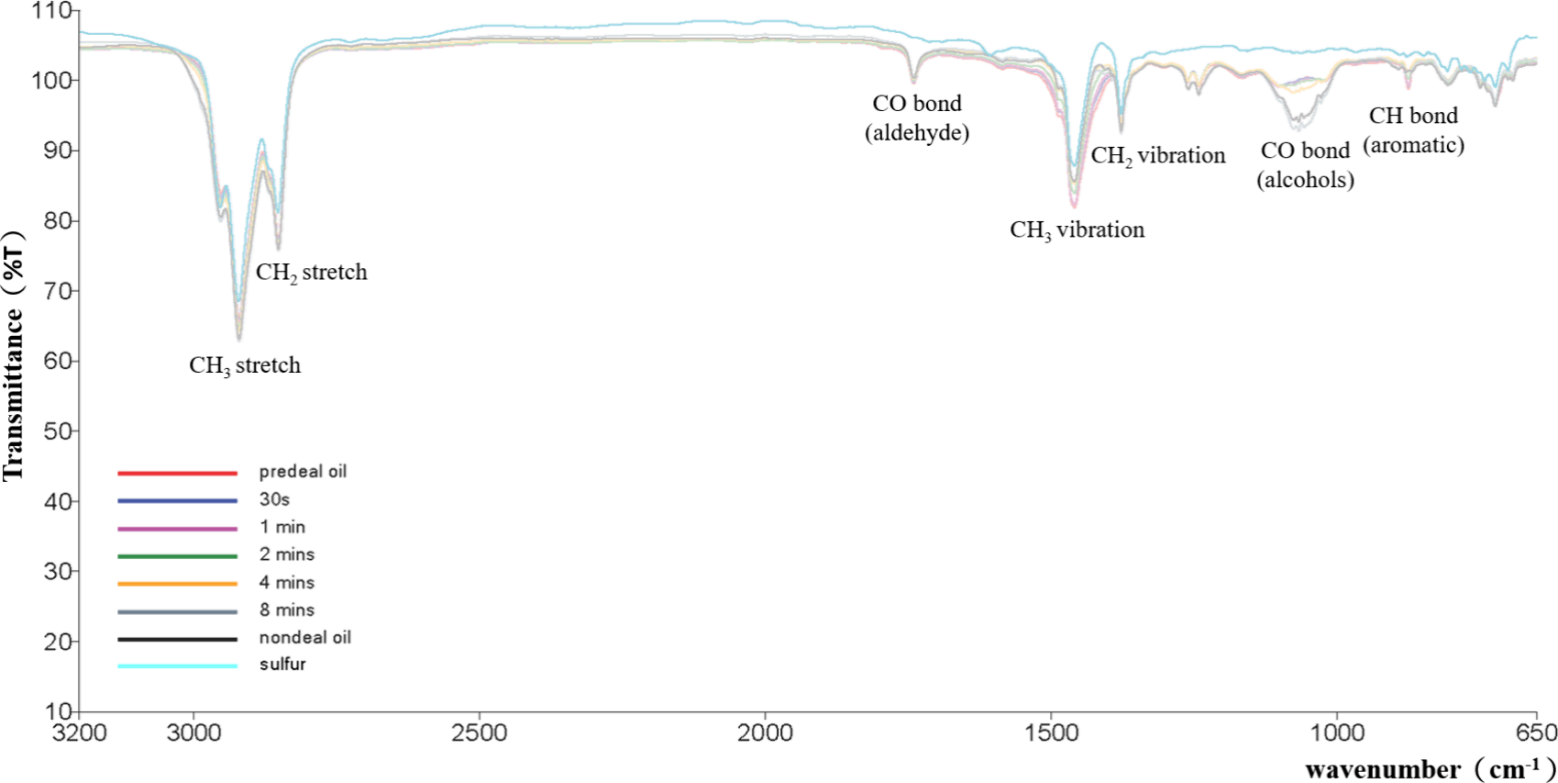
Spectra of the oil after being treated with plasma reaction from Fourier transform infrared spectrum analysis.

There is a rather low sulfur content in the refined oil after being treated by the dielectric-barrier discharge (DBD) plasma. However, the sulfur compounds are distributed widely corresponding to the wave numbers spanned from 3200 to 650 cm^–^^1^, which implies that various structures of sulfur compounds exist in the refined oil. For example, the sulfur compounds of C–S stretching vibrations are generally found at wavenumbers between 600–700 and 2500–2600 cm^–^^1^. The mercaptans (thiols) appear to have a strong S–H stretching band around 2550–2600 cm^–^^1^. The sulfides (thioethers) present C–S stretching vibrations in the 600–700 and 1000–1100 cm^–^^1^ ranges. The thiophene compound due to ring vibrations and C–H out-of-plane bending is observed to have characteristic peaks in the 700–850 cm^–^^1^ region. The other sulfur-containing functionalities may also exhibit absorptions due to sulfones, sulfoxides, and other oxidized sulfur species. Observed from the FTIR results in Figure 8, various structures of sulfur compounds might appear together in the refined oil after being treated with DBD plasma.

### 3.5 Gas Chromatography-Mass Spectrometry (GC-MS) Analysis

Gas chromatography can be used to analyze the components in the mixtures. In this experiment, gas chromatography associated with mass spectrometry was used to analyze hydrocarbons in the refined oil through plasma action. After the sample was evaporated into gas, the inert gas N_2_ was used as a carrier gas transported into a separation column. The retention times were recorded to determine the different chemical compounds.^29^

Figure 9a shows the gas chromatography-mass spectrum obtained from gas chromatography-mass spectrometry analysis, which used commercial petroleum diesel as a fuel sample. It can be seen that commercial diesel contains a series of carbon–hydrogen compounds (C_9_H_20_–C_20_H_42_), mainly included decane (C_10_H_22_), dodecane (C_12_H_26_), 1,3-di*tert*-butylbenzene (C_14_H_22_), and pentadecane (C_15_H_32_). It also contains a small amount of alkenes, aldehydes, and other silane-containing compounds. In contrast, Figure 9b shows the spectrum for the refined oil sample. The refined oil contains a narrower series of carbon–hydrogen compounds (C_14_H_30_–C_20_H_42_). The refined oil sample also contains a wider range of oxygenated compounds of higher concentrations such as alcohols, esters, and aldehydes. Judging from the analysis results in Figure 9, the compositions of the refined oil are consistent with the compounds contained in diesel, which are mainly from carbon 9 to carbon 19 compounds. They are the main component of diesel fuel. While the refined oil also contains a higher concentration of oxygenated compounds, this similarity in its core hydrocarbon structure suggests its potential as a fuel source. Hence, refined oil might be used to replace diesel fuel for vehicles.

**Figure 9:**
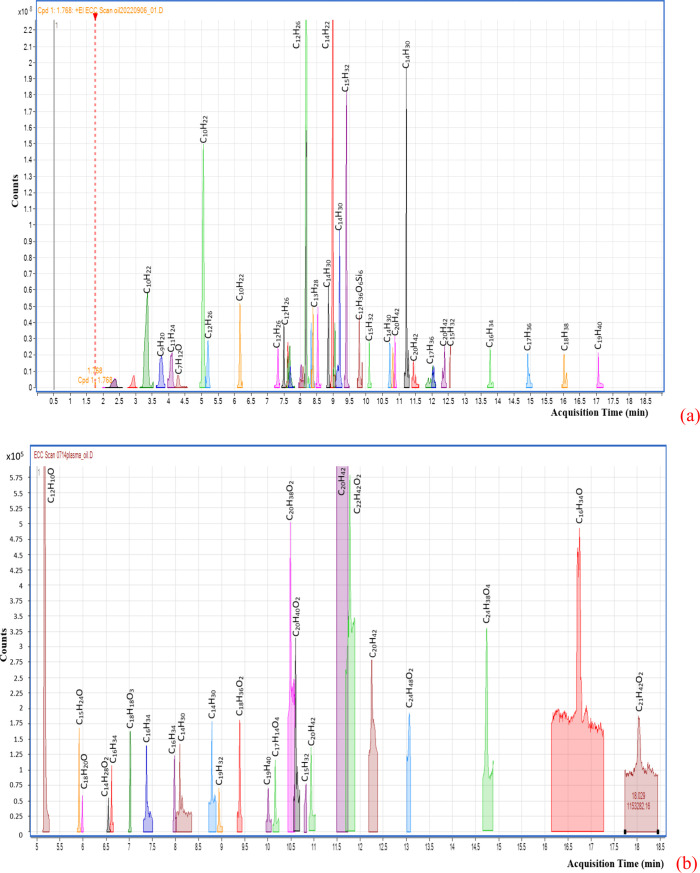
Gas chromatography mass-spectrometry analysis results for (a) commercial petro-diesel; (b) the refined oil through plasma reaction under dielectric particle size 50 μm and the weight ratio of dielectric to sludge equal to 2/1.

## 4 Conclusions

Al_2_O_3_ was used as the dielectric material and the dielectric particle sizes ranged from 10 to 1000 μm, the weight ratio of dielectric Al_2_O_3_ were set at 2/1 and 4/1, and plasma operating time varied from 0.5 to 8 min. The fuel properties of the refined oil through plasma action were analyzed. In the parameter of the weight ratio of dielectric to sludge, it was found that a higher heating value appeared for the weight ratio of dielectric to sludge equal to 4/1 than the weight ratio equal to 2/1 due to the increase in plasma discharge density by the former condition. As the plasma operating time increased, the sludge surface began to carbonize, causing the shrinkage of the plasma operating range to reduce the effect of the plasma on improving fuel quality. Under the same weight ratio of dielectric to sludge and operating time, the dielectric particle size had a decisive impact on the refined fuel properties through plasma action. The smaller the particle size, the superior the refined fuel properties after the plasma operation. For the weight ratio of dielectric to sludge of 2/1 and dielectric particle size of 100 μm, the increase of plasma operating time resulted in the increase of heating value along with the decrease of carbon residue. At the plasma operating time of 8 min, there has the highest heating value and the lowest carbon residue. In contrast, for a weight ratio of dielectric to sludge of 4/1 and dielectric particle size of 10 μm, the highest heating value occurred. The weight ratio of dielectric to sludge of 4/1 and a dielectric particle size of 100 μm caused the highest moisture content of the refined oil. The analysis of Fourier transforms infrared spectroscopy showed that the chemical compounds of the refined fuel are composed of symmetrical CH_3_ stretching, CH_2_ stretching, CH_3_ vibration, CH_2_ vibration, alcohol containing C–O bond, and aromatics containing C–H bond. The gas chromatography-mass spectrum analysis further confirmed that the refined fuel has a similar hydrocarbon structure to diesel, which is composed of C9–C19 compounds. Hence, the refined fuel is expected to be used as an alternative fuel for diesel vehicles.
